# Foreign Notes and Excerpta

**Published:** 1868-09-01

**Authors:** 


### *FOREIGN NOTES AND EXCERPTA.*

M. A. TRIPIER, in a paper read before the Academy of Sciences, Paris, (June 15), suggests the use of anæsthetic inhalation for the relief of every form of hepatic colic. He takes the ground that it is upon the activity of the reflex function that the expulsion of calçuli depends. He further asserts that experiments, in which section of the spinal cord has been practiced, the pathological observations of cerebral paralysis of Marshall Hall, and the results of chloroformic anæthesia in obstetrical practice, demonstrate that the most certain means of increasing the intensity of reflex phenomena is to isolate the organs which constitute its seat, from the controlling influence of the brain. In the case under consideration, the intention is to induce temporary cerebral paralysis, under the influence of which the intensity of reflex phenomena may be increased, and thus the expulsion of the calculi effected.

---

THE exhaustive discussion upon tuberculosis, which has occupied the Academy during so many months, still drags its slow length along. It will undoubtedly develop all that is known upon the subject up to the present date in France.

In view of the marked increase in the prevalence of pulmonary tuberculization in Paris, the medical society of the hospitals of that city has appointed a special commission (*Commission de Phthisiologie*) to pursue the study of this specialty, and has nominated MM. Hérard, president, Chauffard, Moutard-Martin, Villemin, and Potain, secretary. This commission appeals to all physicians to communicate to it all the facts bearing upon the etiology of tuberculosis.

---

DR. LAWSON reports to the *London Lancet* a case of eschar, produced upon the breast of a young woman from the effects of pulverized ether.

---

MESSRS. Protheroe Smith and Heywood Smith, report to the same journal favorably upon the tetrachloride of carbon as an anæsthetic agent, As the result of fifty-one experiments, they recommend its use to relieve pain in Cephalagia, Dysmennorrhœa, Toothache, and that in particular it diminishes pain while it does not diminish the intensity of the contractions of the uterus.

---

MR. BIRKETT (Guy's Hospital), reports the complete reunion of a severed metacarpal bone, by means of wire suture, applied through perforations made in the bone. The same surgeon reports also a case of fractured lower jaw treated successfully in the same way. Also, several cases of ununited fractures of bones, by the introduction of ivory pegs between the fragments.

M. Folker reports to the *Gazette Médicale* a case of poisoning by swallowing three grains of strychnine, which was successfully treated by inhalations of chloroform. This is the second case reported.

---

Trichiniasis and the Trichina is again occupying the attention of the Academy of Sciences (Paris). M. Colin has just presented the results of his investigations into this subject in its relations to Zoology, Hygiene and Pathology. The Hygienic relations of this parasite are the most immediately interesting, and in these nothing specially new is elicited.

---

We learn from Suez that this year the return of the pilgrims from Mecca has been effected in the midst of the most favorable sanitary conditions. The measures adopted by the international conference at Constantinople have operated in the most satisfactory manner.

Seven thousand two hundred and twenty-six pilgrims arrived at Suez, by sea and land. They have been kept in quarantine, and out of the entire number, but twenty-five have died at the lazaretto—twelve of dysentery, eight of pulmonary disease, and five of old age.

---

Biological Society — Paris.— 1. *Physiology :* Changes in the pressure of the air in the chest during the two phases of the respiratory act. By M. P. Best.

During inspiration, a true diminution of pressure exists in the lung, and during expiration a compression of the air. In other words, the calibre of the trachea is not sufficiently large for the demands of the respiratory movements. This is shown by diagrams taken under the following conditions: An animal is placed under a bell glass with tubular attachment closed below, whose capacity is from four to five times the volume of the animal. By means of the tube a communication is established with the drum of Masey's polygraph, the registering cylinder being set in operation, the lever is seen to move in a certain direction at each inspiration of the animal, and then to go in the opposite direction at each expiration succeeding.

The movement following the inspiration indicates an augmentation in the volume of the air contained in the bell. Now this can take place only in consequence of an energetic demand which the animal makes, a demand which is not sufficiently satisfied by the exterior air; so that the air of the lung is dilated again in volume for the system. The contrary takes place from the moment of expiration.

Here is certainly the most simple and the most convenient mode of studying the rythm, and the different conditions of the respiratory movements in animals. It is remarkable that the different animals do not give identical results. Thus a dog, a cat, a rabbit, a duck, all of nearly the same volume, having been placed successively under the same bell, the most emphatic line was that of the duck; then came the dog and the cat, which resembled each other nearly, and after a long interval the rabbit, which made only a great number of minute, rapid oscillations. The same difference was presented by a guinea pig compared with a pigeon. A turtle effects decided movement; an adder and a frog, none at all.

After an interval of some minutes the curve is seen to direct itself in its totality, in a manner which seems to indicate an absorption of the medium: this is due to the insufficient production of carbonic acid, compared to the oxygen supplied. When the animal is excited, the curve is elevated in a manner to indicate a great demand for air, intrathoracic dilatation. This takes place as well from one effort of forcible inspiration, as by a series of profitable (beneficiares) inspirations, that is to say, followed by incomplete expirations. Then comes a series of movements in which expiration gains upon inspiration, which restores the original equilibrium.

It would be interesting to know the value of this diminution of pressure in relation to the quantity of air absorbed at each inspiration, but this experiment could hardly be made upon man.

---

POSITION IN THE REDUCTION OF INGUINAL HERNIA—*By Dr. Joseph B. Bond, Yarmouth, Nova Scotia.*— Several years ago, I had a case in which the patient could not reduce an inguinal hernia while lying in bed, either on his side or on his back, but as soon as he stood on his feet there was not the least difficulty. If, on removing his truss before going to bed, he neglected to apply his hand to the part and allow the rupture to protrude, he had always to get upon his feet before he could reduce it. Soon after this I was called to a case of strangulated inguinal hernia. After making every effort in the usual way to reduce it, I directed the patient to stand up; I placed myself (also standing) behind him, and encircled his body with both my arms, grasped the tumor with both hands, and effected in a few minutes what I had failed to accomplish in as many hours. Since then I have had many cases of inguinal hernia in my own practice, and several where I have been called in consultation, and have never failed to effect a reduction in a few minutes in the way I have described. I have never seen this means tried in the Hospital in Philadelphia nor in the London Hospital, although in both these institutions I have repeatedly seen all efforts fail to reduce an inguinal hernia without an operation. Nor have I ever seen it recommended in any surgical work.

My object in sending you this communication is to ask my medical brethren of the metropolis to give the erect posture in the reduction of inguinal hernia a fair trial, and to publish the results. In femoral hernia the erect posture has never succeeded in my hands — in three cases I have been obliged to use the knife — in inguinal, never. I will not attempt to account for the use of the erect posture in the reduction of inguinal hernia, nor for its failure in femoral. It may be thought that the erect posture favors reduction by causing syncope, but in only two cases do I remember that a feeling of faintness was complained of. In the last case (only a few days ago) the patient, an old man, fainted and fell as soon as the gurgling began to be felt, and I finished the reduction whilst he was prostrate.—*Medical Times and Gazette.*

---

PEROXIDE OF HYDROGEN AS A REMEDY IN DIABETES—*By Dr. John Day, Geelong, Australia.*—[The patient was 36 years of age, and was progressively getting worse, passing as much as five quarts of highly saccharine urine during each night.]

While pondering over the hopeless condition of my patient, it occurred to me that if I could oxidize the sugar that had been taken up in the general circulation, it would be an approach towards the natural mode of elimination by the lungs. With this object in view, I gave half-drachm doses of ethereal solution of peroxide of hydrogen mixed in an ounce of distilled water, three times a day.

To enable me fully to explain the theory on which I base my treatment, would occupy far more of your valuable space than I could justly claim. Schonbein believes that peroxide of hydrogen is H O antozone, and that the blood-corpuscles possess, in a very high degree, the property of decomposing it, and of transforming its antozone into ozone, without, in themselves, undergoing any very rapid change; and he further believes that ozone is the only condition in which oxygen possesses any active combining properties. Assuming these views to be correct, we should possess in ethereal solution of peroxide of hydrogen, which would be rapidly absorbed, a ready means of destroying, by oxidation, the sugar in the blood, and of also maintaining the animal heat, which, in the treatment of diabetes, is an important consideration. I may observe, that what is sold by Mr. Robbins as Dr. Richardson's ozonic ether is, in reality, a solution of peroxide of hydrogen in ether. This may be readily proved by adding a few drops of it to a weak solution of chromic acid: a beautiful blue colour will be the result, caused by the formation of perchromic acid. This preparation is in every respect similar to that which I have been using, and in the therapeutical effects of which I have now had some years' experience.

I commenced the use of this new remedy on August the 10th, and, as the following extracts from my case-book will show, with most gratifying results to the patient:

Aug. 12th. From 10 P.M. to 10 A.M., passed about five pints of urine. Previously for many months, the quantity of urine passed during the night averaged five quarts.

13th. Quantity of urine passed during the night, rather less than three pints and a half. Thirst not so urgent.

14th. Quantity of urine passed during the night, two pints and a half. Urine strongly acid; specific gravity 1046. Thirst much less urgent.

16th. Quantity of urine passed during the night, rather less than forty ounces. The patient very much improved in every respect. I give her own words:—"I have no thirst now; no more than I had in olden times. I feel that I am cured if it will only last."—*Lancet.*

---

Carbolic Acid a Cure for Toothache.—Among the many virtues of Carbolic acid is that of giving relief from the pain of toothache. I have tried it in a great many cases, and with invariable success. To one drachm of collodium flexile (B. P. 1867) add two drachms of Calvert's carbolic acid, full strength. A gelatinous mass is precipitated. A small portion of this precipitate inserted into the cavity of an aching tooth gives immediate relief. It may be kept in the cavity by means of a bit of lint dipped in the collodium.—*Lancet, Feb.* 22, 1868, *p.* 275.

---

*To the Editor* Chicago Medical Journal—*Dear Sir:* Will you kindly insert the following notice or its equivalent in your paper, and thus aid a worthy cause?

The New York Medical College for Women will begin their Sixth Annual Term of twenty weeks, at their new College in Twelfth Street, corner of Second Avenue, the first Monday in November. For announcements, giving full particulars, address, with stamps, the Dean, Mrs. C. S. Lozier, M.D., or the Secretary, Mrs. C. F. Wells, Box 730, N. Y.

Comments are unnecessary.—Ed.

